# Existence of life-time stable proteins in mature rats—Dating of proteins’ age by repeated short-term exposure to labeled amino acids throughout age

**DOI:** 10.1371/journal.pone.0185605

**Published:** 2017-09-28

**Authors:** Cecilie Leidesdorff Bechshøft, Peter Schjerling, Andreas Bornø, Lars Holm

**Affiliations:** 1 Institute of Sports Medicine, Department of Orthopedic Surgery M, Bispebjerg Hospital and Center of Healthy Aging, Faculty of Health and Medical Sciences, University of Copenhagen, Copenhagen, Denmark; 2 Clinical Metabolomics Core Facility, Department of Clinical Biochemistry, Rigshospitalet, Copenhagen, Denmark; 3 Institute of Biomedical Sciences, Faculty of Health and Medical Sciences, University of Copenhagen, Copenhagen, Denmark; Pacific Northwest National Laboratory, UNITED STATES

## Abstract

In vivo turnover rates of proteins covering the processes of protein synthesis and breakdown rates have been measured in many tissues and protein pools using various techniques. Connective tissue and collagen protein turnover is of specific interest since existing results are rather diverging. The aim of this study is to investigate whether we can verify the presence of protein pools within the same tissue with very distinct turnover rates over the life-span of rats with special focus on connective tissue. Male and female Lewis rats (n = 35) were injected with five different isotopically labeled amino acids tracers. The tracers were injected during fetal development (Day -10 to -2), after birth (Day 5–9), at weaning (Day 25–32) at puberty (Day 54–58) and at adulthood (Day 447–445). Subgroups of rats were euthanized three days after every injection period, at different time point between injection periods and lastly at day 472. Tissue (liver, muscle, eye lens and patellar tendon) and blood samples were collected after euthanization. The enrichment of the labeled amino acids in the tissue or blood samples was measured using GC-MS-MS. In muscle and liver we demonstrated a rapid decrease of tracer enrichments throughout the rat’s life, indicating that myofibrillar and cytoskeleton proteins have a high turnover. In contrast, the connective tissue protein in the eye lens and patellar tendon of the mature rat showed detainment of tracer enrichment injected during fetal development and first living days, indicating very slow turnover. The data support the hypothesis that some proteins synthesized during the early development and growth still exist much later in life of animals and hence has a very slow turnover rate.

## Introduction

To allow growth and remodeling as well as functional integrity most proteins are undergoing a process of continuous turnover involving degradation of existing proteins and biosynthesis of replacement proteins. Synthesis and breakdown rates of proteins have been measured in many tissues and protein pools using various techniques and half-lives ranging from few hours to months/years have been found in larger animals and humans. Connective tissue and collagen protein turnover is of specific interest, since existing results on synthesis (and turnover) rates are conflicting. Some studies indicate that matrix collagen proteins synthesized early in life in humans are preserved into adulthood in tissues like eye lenses and tendon [1, 2]. In contrast, other studies utilizing other techniques report that collagen protein in human tendon and muscle has a synthesis rate in adulthood [3] and in older age [4, 5] that is comparable to e.g. myofibrillar proteins from skeletal muscle.

Specifically in relation to collagen protein the turnover rates can be determined by utilizing different methodological approaches and principles. For example, several possibilities exist to measure changes in concentrations of biomarkers referring either to the synthesis or breakdown processes of collagen. One is the posttranslational hydroxylation of proline residues to hydroxyproline in collagen. Since hydroxyproline is not reutilized for protein synthesis it is disposed to the plasma and excreted in the urine upon degradation of collagen proteins [6]. Therefore, the amount of hydroxyproline in the blood and/or urine is used to reflect collagen protein degradation [6, 7]. Similarly, abundance of hydroxyproline with microdialysis technique has been demonstrated in human skeletal muscle [8] and thereby collagen degradation has been shown.

Other types of assessments of collagen protein turnover involves the measurement of abundances of biomarkers such as pro-collagen type I C-terminal peptide (PICP), pro-collagen type I N-terminal peptide (PINP), COOH-terminal telopeptides of type-I collagen (CTX-I) and C-terminal type II pro-collagen peptide (pCOL-II). These biomarkes are cleaved of the pro-collagen during synthesis (PICP, PINP and pCOL-II) or formed during breakdown of the mature collagen (CTX-I). The above mentioned biomarkers have been used extensively to demonstrate rather large fluctuations in collagen turnover in human bones, tendons, ligaments and cartilage, where they are determined in interstitial peritendinous fluid, joint fluids as well as measured in plasma and urine dependent on tissue origin [9–11]. Differences in the appearance rate and/or concentration of peptides have been used as a single-point measurement to demonstrate changes in collagen turnover in the early recovery after e.g. exercise indicating a relative fast turnover of collagenous proteins [9, 10].

Another protein turnover assessment principle is the direct tracer incorporation technique to assess the synthesis rate of newly synthesized proteins. By injection/infusion of a stable isotopically labeled amino acid tracer it will enter the precursor pools for protein synthesis, label the tRNA and thereby be incorporated into newly synthesized proteins [12]. Using this methodology the direct measure is on the proteins isolated for analysis of tracer abundance. With such protocol, collagen protein synthesis rates have been measured in various tissues; in human skeletal muscle rates between 0.04%/h [3] and 0.08%/h [13, 14] have been observed dependent on specimen preparation procedure. In human tendon tissue rates of 0.05%/h [3] down to 0.01%/h [15, 16] have been observed.

Yet another approach to study protein turnover is to estimate the age of the proteins. Investigating the accumulation of the D-form amino acids in a protein, called racemization, can do this. Racemization is the random process of amino acids changing from their natural isomeric L-form to D-form after being incorporated into proteins with a rate of about 1/1000 per year [17]. This technique has been used in horses to demonstrate that rather old collagenous protein exists in some tissues and that collagen in a tendon turns over in approximately 200 years [18], which is indeed very slow compared to rates measured by other methods mentioned, using the biomarker approaches showing changes in hours, though in humans [9, 13, 14].

Another sophisticated method to study slow turning over proteins is the application of the bomb pulse dating: as a consequence of the atmospheric nuclear-bomb testing in the period 1955–1963, there was a dramatic rise in the atmospheric level of ^14^C. The ecological utilization and recirculation of carbon made all organisms (from plants taking up the atmospheric CO_2_ to herbivores and finally carnivores) to reflect the exiting level of atmospheric ^14^C in the protein at the time of their synthesis. The ^14^C eventually ended up in human tissue as well [19] and its presence in different tissues such as the eye lens and tooth enamel has then been used to determine the age of human bodies after decease [1, 20]. The bomb pulse dating was furthermore used by Heinemeier et al. showing that proteins derived from tendons have a similar slow turnover as the eye lens and tooth enamel [2] in line with the racemization data from horses but very distinct from the turnover rates measured using other methodologies.

It is therefore very inconclusive how fast the collagenous proteins in various tissues turn over. However, all these techniques measure average protein turnover rate, so the apparently conflicting results could be explained by assuming that one fraction of the proteins in the measured pool has a very high turnover and another fraction a very slow turnover. Acute measures like breakdown products and direct tracer incorporation over few hours would then mainly measure the fast turning over pool, whereas long-term techniques like racemization and bomb pulse dating would measure the pool with a slow turnover.

The primary aim of the present study is to investigate the turnover of collagenous tissue proteins throughout life. With the use of stable isotopically labeled amino acids (hereafter referred to as tracers) early in life, we want to verify the presence of protein pools in different tissues and within the same tissue with very distinct turnover rates and whether other amino acid tracers given at older age are incorporated into tissue structures. We use a rodent in vivo model and focus on structural proteins, especially collagenous connective tissue, since these are expected to be the proteins with the slowest turnover rate. We sat up an experiment where rats are given repeated injections of different amino acid tracers for short periods of time at different ages. We hypothesize that during these short time-slots at various ages, the different amino acid tracers will be incorporated into any protein synthesized at this time and upon a very slow turnover the tracers will be present in the proteins later in life.

## Materials and methods

### Experimental design

Rats were exposed for different amino acids tracers for a short period of time at different ages. During exposure, the amino acid tracers were incorporated into proteins that were synthesized at the time of tracer appearance and thereby labeling the newly synthesized protein.

14 female and 7 male Lewis rats each 9 weeks old (Charles River, Sulzfeld, Germany) were used as parent rats, thus mated and the mothers gave birth to puppies that subsequently were used for the experiment.

### Animals

Rats were housed with 2–3 rats per cage at the animal facility (The Campus Stable, University of Copenhagen) and fed chow and water ad libitum by staff at the animal facility. The rat cages were maintained at: 22°C (± 2°C), humidity of 55% (± 10%) and at a 12:12 hour light/dark cycle. The study was approved by the Animal Experiments Inspectorate of the Danish Ministry of Food, Agriculture and Fisheries, approval ID: 2012-15-2943-00073.

### Tracer injection protocol

The tracer rats were exposed to up to five different tracers during the 472 days referring to five different ages: Fetus: U-^13^C_5_-proline (Fet-Pro) (Day -10, injection to the mother rats every second day prior to delivery (defining Time point 0 of the tracer rats)); Pup: ring-^13^C_6_-phenylalanine (Pup-Phe) Day 5 (one injection daily for five consecutive days starting at the age of five days); Weaning: 1,2-^13^C_2_-leucine (Wea-Leu) Day 25 (one injection daily for five consecutive days starting at the age of 25 days); Puberty: ^15^N-proline (Pub-Pro) Day 54 (one injection daily for five consecutive days starting at the age of 54); and Adult: 1,2-^13^C_2_-glycine (Adu-Gly) Day 447 (one injection daily for five consecutive days starting at the age of 447 days). The days where each tracer is injected will from here on be termed ‘tracer period’.

**Fig 1:**
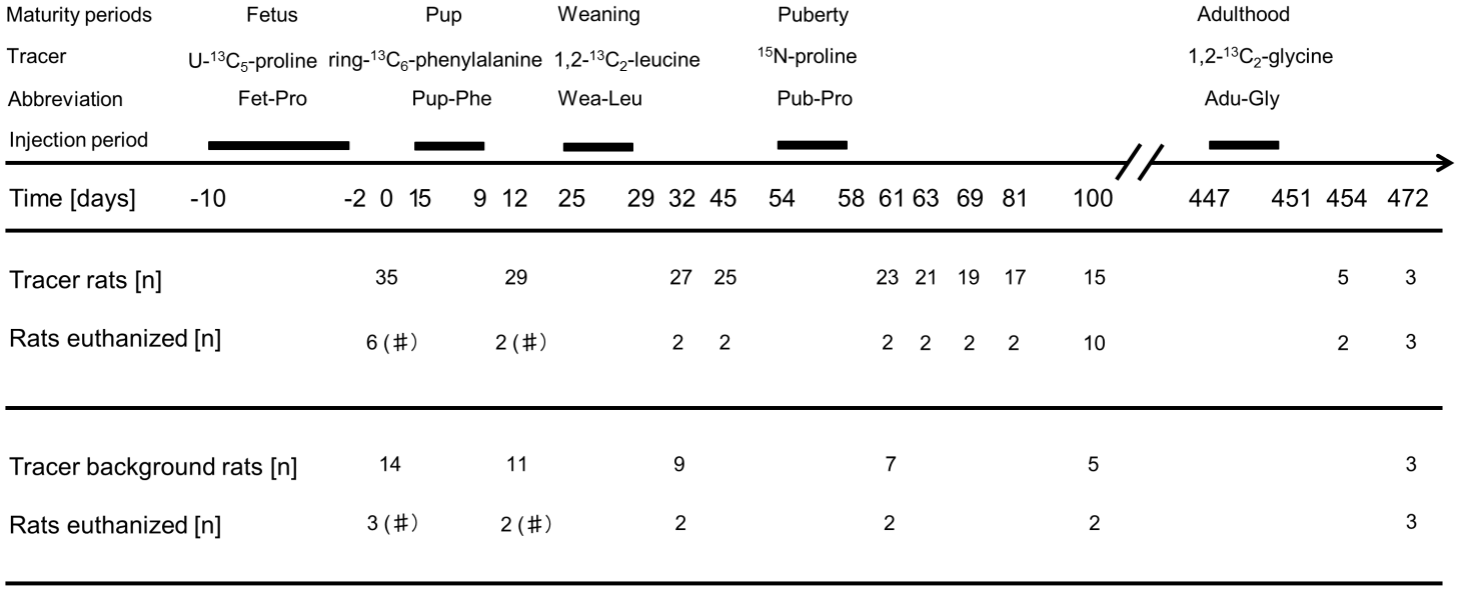
Tracer injection protocol. Lewis rats n = 35 were injected with up to five different tracers (tracer rats). The tracers were injected during the rats’ fetal development (days -10 to -2, with tracer U-^13^C_5_-proline, Fet-Pro), after birth (days 5–9, with tracer ring-^13^C_6_-phenylalanine, Pup-Phe), at weaning (days 25–32, with tracer 1,2-^13^C_2_-leucine, Wea-Leu), at puberty (days 54–58, with tracer ^15^N-proline, Pub-Pro) and at adulthood (days 447–451, with tracer 1,2-^13^C_2_-glycine, Adu-Gly). The tracers were injected once a day for five consecutive days, except during the fetal development, where injections were done every second day during the second half of the pregnancy of the mother rat. A subset of rats was euthanized on day 1, 12, 32, 45, 61, 63, 69, 81, 100, 454 and 472, after last of the previous injections and on days in between. This was done in order to verify the incorporation of amino acid tracers into the fast turning over proteins. Furthermore, n = 14 rats that didn’t receive any tracer injections (tracer background rats) were euthanized on some of the same days as the tracer rats (day 1, 12, 32, 61, 100 and 472). Only mixed muscle and liver samples are collected from euthanized rats from day 1 and 12 (marked by a hash tag).

For a schematic overview of the experimental protocol, see Fig 1. The experiment is described in more detail below.

Fourteen mother rats were housed with a father rat for 48 hours to mate. Pregnancy was confirmed by external uterus palpation in the last part of the gestation period termed day -10. Upon pregnancy, twelve of the mother rats were anaesthetized with isoflurane gas (1.0 L/min oxygen with 3.5% isoflurane Baxter) and injected intraperitoneally with tracer Fet-Pro on days -10, -8, -6, -4 and -2 in the gestation period. The remaining two mother rats did not receive any tracer injections.

The day of birth of the rat puppies was defined as day 0 and 35 puppies of both female and male sex (tracer rats) were born and included in the experiment. Additional 14 rat pups (tracer background rats) were born by non-injected mother rats. These were used for determination of natural abundance of the tracers at corresponding time points to the tracer rats.

On day 1, six of the thirty-five tracer puppies were euthanized and tissue was collected.

On days 5, 6, 7, 8, and 9, twenty-nine tracer puppies were intraperitoneally injected with tracer Pup-Phe. On day 12 (three days after the last injection of tracer Pup-Phe) two rats were euthanized, and blood and tissues were collected. Injections of the remaining tracer rats were repeated on days 25–29 (n = 27, weaning age, tracer Wea-Leu), days 54–58 (n = 23, puberty age, tracer Pub-Pro) and days 447–451 (n = 5, adulthood, tracer Adu-Gly) and two days after the last injection in each period two rats were euthanized to verify the incorporation and presence of amino acid tracer in fast turning over proteins. Furthermore, two tracer rats were euthanized on day 45, 63, 69 and 81, in order to follow and examine tracer disappearance from the rat body. On day 100, when the rat was considered mature, ten tracer rats were euthanized to investigate presence of the first four tracers in different tissues. Five tracer rats continued to the fifth tracer injection period on day 447–451. Two tracer rats were euthanized two days after the fifth injection period, and the last three rats were euthanized 23 days after, on day 472.

The tracer background rats did not receive any tracer injections, but were euthanized and tissue collected at same ages as the tracer rats: three on day 1, two on day 12, 32, 61, and 100, and three on day 472.

It was originally planned to finalize the experiment at day 100, therefore twelve rats were euthanized (tracer rats n = 10, tracer background rats, n = 2), but it was decided to continue the experiments with eight rats (tracer rats n = 5, and tracer background rats n = 3) to day 454. At day 454 two tracer rats were euthanized and the last three tracer rats continued to day 472. The experiment was prolonged in order to further investigate whether amino acid tracers could be incorporated into the various proteins in the aging rat.

### Stable isotope amino acids tracers

All five stable isotopically labeled amino acid tracers (sterility and pyrogenity tested, 99% enriched) were purchased from Cambridge Isotope Laboratories (Andover, MA, USA) and injected at different time points in the rats’ life in the following order: Tracer Fet-Pro: (U-^13^C_5_-proline), Tracer Pup-Pro: (ring-^13^C_6_-phenylalanine), Tracer Wea-Leu: (1,2-^13^C_2_-leucine), Tracer Pub-Pro: (^15^N-proline), and Tracer Adu-Gly: (1,2-^13^C_2_-glycine).

Stock tracer solutions of even amounts of tracers per body weight (approximately 100 μmol tracer/100 g body weight (BW) hence, not taking differences in basal concentrations of the three tracees into account) were prepared prior to the execution of the study. The BW of the rats at any ages of the injection was estimated at the time of preparation and the tracer amounts were solubilized into sterile isotonic 9 g/L saline, sterilized using a sterile nonpyrogenic 0.20 μm syringe filter (Satorius Stedim Biotech, Germany), and kept at 5°C until injection day. Just before injection, the tracer was again sterilized through a 0.20 μm syringe filter. The injections were performed intraperitoneally as a single bolus at each of the specified days.

### Sample collection and storage

Blood samples were collected to verify tracer enrichment in the circulation at various time points during the period of injections. Each rat was anaesthetized with isoflurane gas (1.0 L/min oxygen with 3.5% isoflurane Baxter), and blood samples were drawn from the jugular vein. Tracer injections were given once per day for five days, and blood samples were collected three times during the injections period. A blood sample was collected before the first injection, again three hours after first injection and the third on the fifth day of injections approximately 24 hours after the fourth injection and just before the fifth injection. This blood-sampling schedule was applied in each of the five injection periods in the experiment in order to describe the abundance of the free tracers in the blood in each of the tracer periods.

All blood samples were collected in Eppendorf vials, kept on ice for at least 30 minutes, and spun (3172 g, 4°C, 10 min). Serum was transferred to a new Eppendorf vial and kept at -80°C until further analysis.

For tissue collection the rat was euthanized using a subcutaneous injection of Hypnorm/Midazolam (25% of a mixture of 0.315 mg/mL fentanyl and 10 mg/mL fluanisone; 25% of 5 mg/mL Midazolam and 50% sterilized water; injected 300 μL/100 g BW) followed by a thorax rupture, heart puncture and blood sampling directly from the heart. Afterwards, liver (midlobe), patellar tendon, skeletal muscle sample (quadriceps (hereafter referred to as mixed muscle) or soleus muscle) and the eye lens were dissected as fast as possible, cleaned for blood, frozen in liquid nitrogen and stored in -80°C until further analysis. Not all tissues were collected at every time point due to the size and the anatomical development of the rats (on rats born from day 1–32 a specimen of the quadriceps muscle was dissected because the soleus muscle could not be identified (marked by a hash tag on Fig 1); eye lens on rats born from day 1–32 were not collected since it could not be identified; patellar tendon on rats born from day 1–32 were not collected since it could not be identified).

### Protein incorporation

#### Tissue fractions

An amount of approximately 10 mg (patellar tendon ranging from 2.6–16.6 mg; eye lens 4.7–40.9 mg) or 30 mg tissue (liver 24.5–31.8 mg; soleus muscle 8.5–31.6 mg; mixed muscle 19.0–30.4 mg) was used for protein fractioning. The tissue was homogenized with eight Lysing matrix D Bulk ceramic beads (MP Biomedicals, Santa Ana, CA, USA) and two 1.0 mm silicon carbide beads (Bio Spec Products Inc, USA) in a 1.0 mL 5°C homogenization-buffer solution (0.02 M Tris, 0.15 M NaCl, 2mM EDTA, 2 mM EGTA, 0.5% Triton-X, pH 7.4) using a FastPrep-24 homogenizer (Thermo Savant, Holbrook, NY, USA), left at 5°C for 3 hours and then spun (800 g, 20 min, 5°C).

The supernatant containing the cytoplasmic protein fraction was transferred to new vials and saved for potential future but was not analyzed. The pellet from the first spin was washed with 1.0 mL homogenization-buffer solution and left at 5°C for 30 minutes, and then spun (800 g, 20 min, 5°C). The supernatant was discarded. The pelleted proteins were separated in a salt solution, to get a salt soluble fraction containing mainly the cytoskeletal/contractile protein fraction (hereafter referred to as CYTO) and a salt insoluble fraction containing mainly the extracellular matrix proteins (connective tissue and collagen) fraction (here after referred to as CT) by adding 1.5 mL high salt buffer (0.7 M KCl, 0.1 M Na_4_P_2_O_7_) left at 5°C over-night and spun (1600 g, 20 min, 5°C) the next day. The supernatant was transferred to new vials and proteins were precipitated by adding 2.3 x supernatant volume of 99% ethanol, left at 5°C for two hours and spun (1600 g, 20 min, 5°C). The supernatant was discarded and pellet was washed with 1.0 mL 70% ethanol, left at 5°C for 30 minutes and spun (1600 g, 20 min, 5°C). The supernatant was discarded and the pellet was stored at -20°C until further analysis. This pellet contained the salt soluble protein fraction and thus the CYTO.

The pellet from the spin in the high salt buffer was washed with 1.0 mL high salt buffer and left at 5°C for two hours, and afterwards spun (1600 g, 20 min, 5°C). The supernatant was discarded and the pellet was washed with 1.0 mL 70% ethanol, left at 5°C for 30 minutes and spun a last time (1600 g, 20 min, 5°C) to get the connective tissue pellet fraction and thus the CT.

All proteins pellets were hydrolyzed with 1.0 mL 6 M HCl at 110°C for more than 16 hours before further processing of the constituent amino acids.

#### Serum fractions

Serum samples were divided into a blood protein fraction and free amino acid fraction. 200 μl serum was added 1.0 mL 2% perchloric acid (PCA), mixed and left at 5°C for 30 min and spun (3172 g, 4°C, 10 min) to precipitate the blood proteins. The free amino acids from the supernatant were hereafter purified (described next section). The blood protein pellet was hydrolyzed by 1.0 mL 6 M HCl at 110°C for more than 16 hours, before further processing.

#### Amino acid purification and derivatization

Amino acids from different tissue and serum fractions were purified by a cation exchange resin column (AG 50 W-X8-Resin, Bio-Rad Laboratories, Hercules, CA, USA). The resin was acidified with 50% acetic acid before sample application to allow the amino acids in samples to bind to the resin. The hydrolyzed fraction was added to the resin column and washed five times with 2 mL Millipore H_2_O. Afterwards the amino acids were eluted from the resin by adding 2 x 1 mL 2M NH_4_OH, making the column alkaline. The eluate was evaporated under a stream of nitrogen, making the amino acids ready for derivatization. All samples were derivatized by adding equal amounts of N-methyl-N-(tert-butyldimethylsilyl) trifluoroacetamide (MTBSTFA) + 1% tert-butyl-dimethylchlorosilane (t-BDMCS) (Regis Technologies Inc., IL, USA) and acetonitrile and diluted for optimal signaling. Hereafter, vortex mixed and heated for 70°C for 1 hour, vortex mixed again and transferred to vials.

#### Determination of tracer enrichment, mass spectrometry (MS)

Tracer enrichment was determined in the fractions CYTO and CT in the muscle and liver. Since the eye lens and patellar tendon mainly consist of collagen, only the CT fraction was analyzed of tracer enrichment.

The tracers were analyzed by gas chromatography-tandem mass spectrometry (GC-MS-MS) in three analysis runs; 1,2-^13^C_2_-glycine was analyzed in a single run; ring-^13^C_6_-phenylalanine and 1,2-^13^C_2_-leucine in another run and ^15^N-proline and U-^13^C_5_-proline in a third run.

On the GC-MS-MS, 0.5–1.0 μL sample was injected (inj. speed 50 μL/s) using a TriPlus Autosampler (Thermo Scientific, Milano, Italy). The inlet temperature was 50–60°C; the split flow was 50 mL/min, and a splitless time of 5 min was utilized. The inlet was operated in Programmed Temperature Vaporizing (PTV) solvent split mode with an injection flow of 30–50 mL/min, the injection transfer temperature was 220–230°C, and the transfer time was 5 min (an evaporation phase was utilized for proline, 60°C [held for 1 min]). The injector was cleaned at 300°C [held for 10 min] with a flow of 200 mL/min. The injector needle was cleaned in acetonitrile.

In the Trace GC Ultra oven (Thermo Scientific, Milano, Italy) the following temperature gradient was used for 1,2-^13^C_2_-glycine measurements: initial temperature 50°C [held for 1 min], slow ramp [20°C/min] to 190°C, fast ramp [50°C/min] to 300°C [held for 10 min]. For ring-^13^C_6_-phenylalanine the temperature gradient was: initial temperature 50°C [held for 1 min], ramp [20°C/min] to 300°C [held for 10 min]. For proline measurements the temperature gradient was: initial temperature 50°C [held for 1 min], 1.st ramp [20°C/min] to 170°C, 2.nd ramp [4°C/min] to 210°C, 3.rd ramp [20°C/min] to 300°C [held for 10 min]. The gas saver flow was 10 mL/min and the gas saver time was 19–25 min. A GC capillary column (CP-Sil 8 CB low bleed 30 m x 0.32 mm, coating 0.25 μm, ChromPack, Varian, Palo Alto, CA) was used and helium was used as carrier gas.

More specifically GC-MS-MS measurements were performed on a triple stage quadrupole mass spectrometer, TSQ Quantum (Thermo Scientific, San Jose CA, USA) operated in electron ionization (EI) mode. The general settings for the mass spectrometer were: positive polarity, profile mode and emission current 50 μA.

Glycine and 1,2-^13^C_2_-glycine were measured in Q3 scan mode with the following settings: scan range 244.00–249.50 mass-to-charge (m/z) units and scan time 0.115 s. Leucine, 1,2-^13^C_2_-leucine, phenylalanine and ring-^13^C_6_-phenylalanine were measured in a combined run. Settings were: scan range 301.00–309.00 m/z and neutral loss mass 132.00 m/z for leucine and leucine tracer and scan range 233.00–244.00 m/z and neutral loss mass 56.00 m/z for phenylalanine and phenylalanine tracer. Other settings were: scan time 0.1 s, collision energy 10 V and collision gas (Ar) pressure 0.7 mTorr.

Proline were measured in a run using the following selected reaction monitoring (SRM) scans: 286.15 → 258.15 m/z (proline); 287.15 → 259.15 m/z (^15^N-proline); 291.15 → 262.15 m/z (U-^13^C_5_-proline), scan time 0.033 s collision energy 10 V collision gas (Ar) pressure 0.7 mTorr.

Data processing including peak areas and tracer enrichment determinations were carried out by MassRatio version 4.15 (FBJ Engineering; Denmark).

### Statistics

Values are presented as enrichment values calculated as a Tracer to Tracee Ratio (TTR), where the natural isotope ratio of the different tracers measured in the tracer background rats are subtracted from the measured isotope ratios in the tracer rats and shown as individual values or mean ± 95% confidence interval. An unpaired two sample t-test (one-tailed, only increase) between tracer and tracer background rats was performed on data collected from day 100 (n = 9–10) and values of *p* < 0.05 were considered significant. All other time points with few tracer rats (n = 2–6) are illustrated as individual values and no statistics have been applied.

## Results

**Fig 2:**
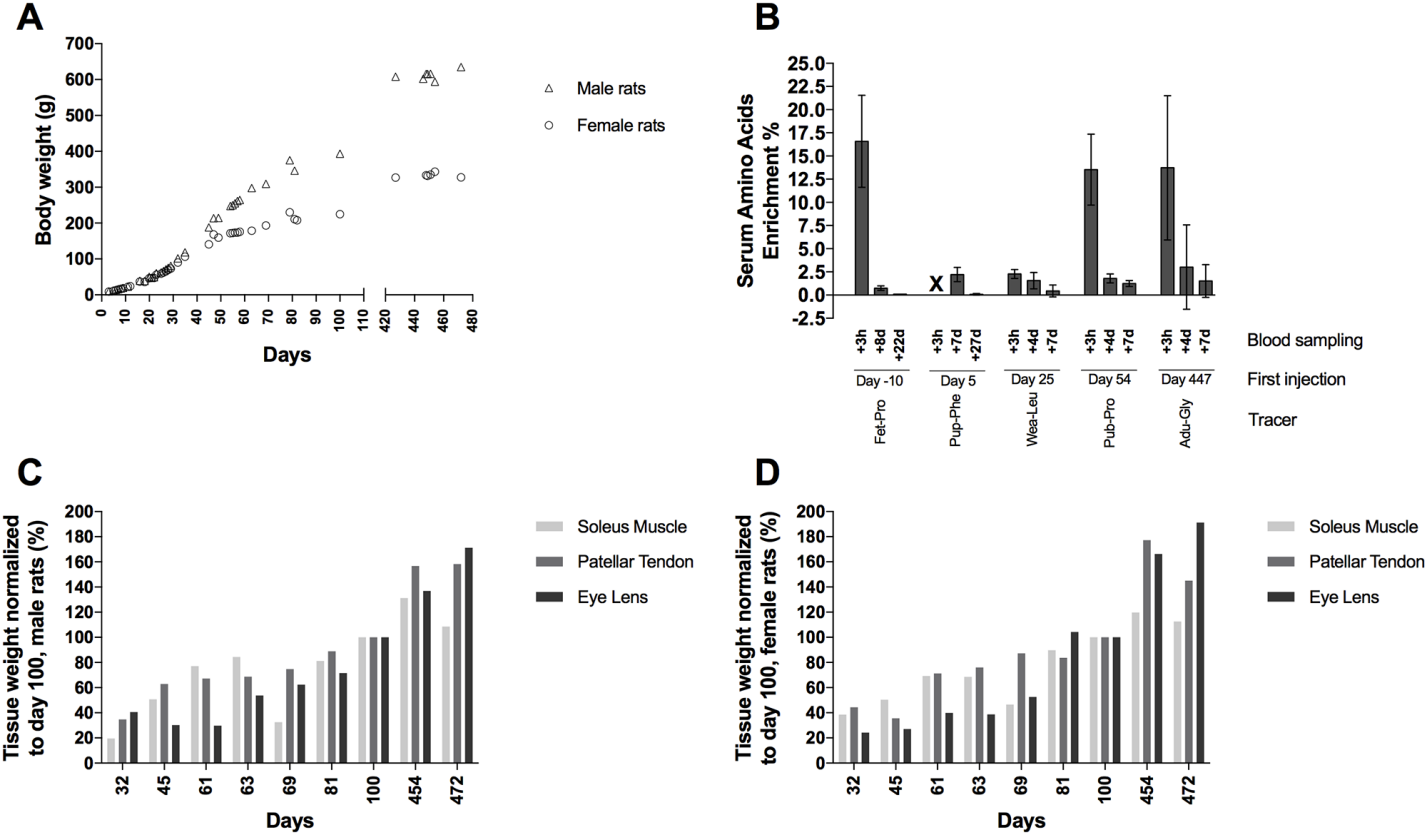
Body weight, tracer verification and tissue weight. (A) Mean bodyweight (g) from day 0–472 in male (triangle) and female rats (circle), (B) Tracer enrichment (%) in amino acids collected from serum samples from injected rats at three hours after the first tracer injection for that specific tracer/age (blood sampling +3h), and at one day after the fourth injection and immediately prior to the last/fifth injection (blood sampling +4d) and at a later day: (+22d, +27d, +7d) after the first injection for tracer rats. +3h for Pup-Phe is not measured (X). Values are represented as mean ± 95% CI, (C-D). Relative tissue weight of the soleus muscle, patellar tendon and the eye lens from day 32–472 in male (C) and female (D) rats (% of day 100) (n = 1, except n = 5 for day 100) normalized to day 100.

A total of 49 rats (35 tracer rats and 14 tracer background rats) were included in the experiment including rats living to the age of 472 days. The rats were euthanized on specific time points during the experiment (see Fig 1). The rats gained weight over the entire period (Fig 2A) and the last rats were euthanized at a weight of 635 g for the male rat and weights of 302.5 g and 352.5 g for the two female rats.

Fig 2C and 2D show the relative weight of the collected tissues; soleus muscle, patellar tendon and the eye lens in the male and female rats. The weight of the tissues is normalized to the mean tissue weight at day 100 (reference age, because tissues from 10 rats were analyzed at this age). The figures show that all the tissues increase in their weight along with the rats’ increase in body weight. The increase in tissue and body weight will have an impact on the results. Even, if the absolute amount of incorporated tracer remains the same during the experiment, then, due to the tissue growing in size, the tracer will be diluted and the results will therefore show a decrease in enrichment.

### Injection of tracers

During the life-span, the rats were injected with five different tracers (Fig 1), each over a five day period (exception was over ten days during fetal development). To test serum enrichment, a blood sample was drawn three hours after the first tracer injection (+3h), and a second was drawn four days later just before the last tracer injection (+4d) and finally a third blood sample, seven days after the first injection (+7d). Fig 2B shows the mean free serum amino acid enrichment (%) of all five tracers during and shortly after their respective injection periods. Exceptions to this blood sample schedule are: the first tracer (Fet-Pro) (exposed to the tracer rats during the fetal development) was injected in the mother rats every second day, hence, blood sampling +8d is just before the last injection but two days after the most recent injection, and blood sampling +22d corresponding to the age of 12 days for the puppy rats. Pup-Phe data for +3h and +4d are missing due to inability to draw blood from the small pups (another blood sample was drawn at +27d, hence 32 days for the puppy rats).

All measured tracers were abundant in the blood three hours after injection with an approximate mean enrichment of 15%. The Wea-Leu tracer was an exception. We had problems in getting the leucine tracer dissolved fully during preparation and we probably lost a fraction in the filtration process. Hence the Wea-Leu tracer only reached an enrichment of 2.28% at +3h after injection time point. At later time points after the injections all the enrichments were markedly lowered.

### Tracer enrichment from day 1–472 in various tissues

#### Liver

**Fig 3:**
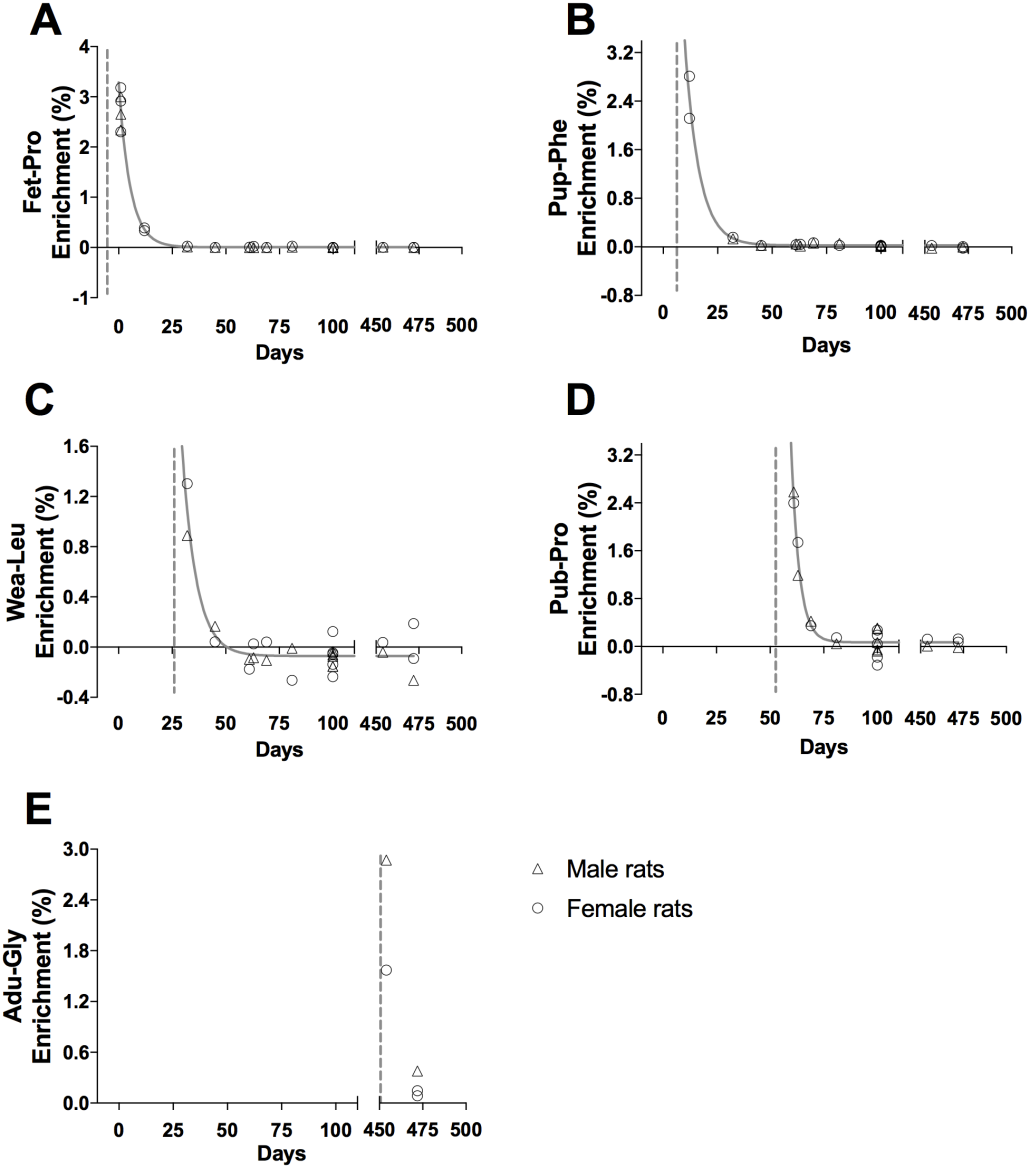
Tracer enrichment in liver cytoskeletal proteins, CYTO. (A) Fet-Pro, (B) Pup-Phe, (C) Wea-Leu, (D) Pub-Pro, (E) Adu-Gly. Male (triangle) and female rat (circle) values are represented as individual enrichment (%) values in the liver CYTO. The dashed vertical line indicates the start of the injection period of the tracer. An exponential decay regression curve is marked by a grey line in A—D (t_1/2_: (A) 3.8 days, (B) 4.6 days, (C) 4.6 days and (D) 2.6 days).

**Fig 4:**
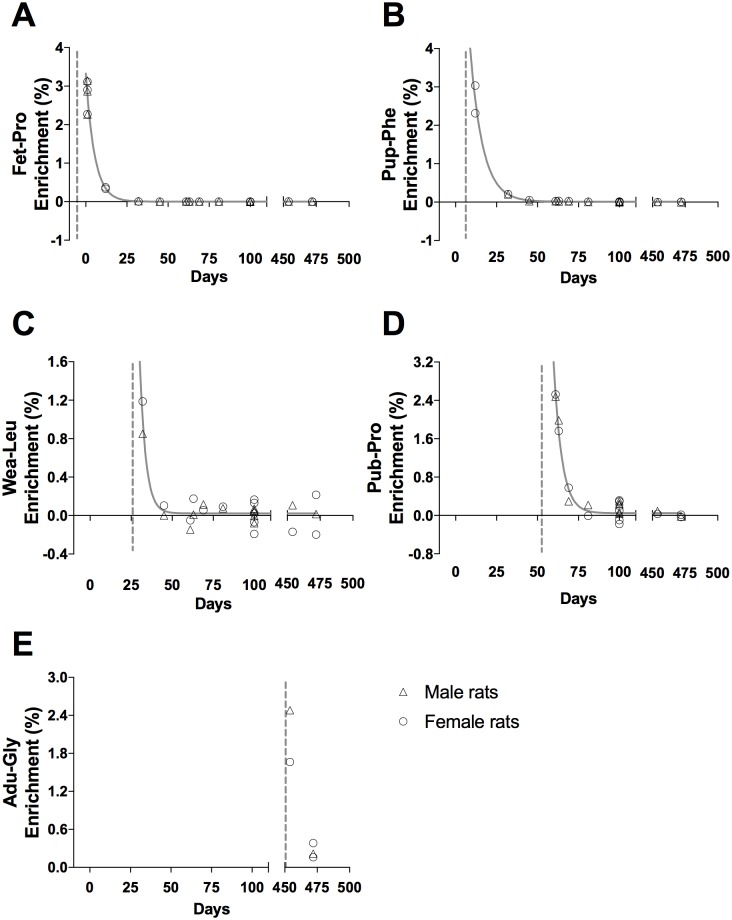
Tracer enrichment in liver connective tissue proteins, CT. (A) Fet-Pro, (B) Pup-Phe, (C) Wea-Leu, (D) Pub-Pro, (E) Adu-Gly. Male (triangle) and female rat (circle) values are represented as individual enrichment values (%) in the liver CT. The dashed vertical line indicates the start of the injection period of the tracer. An exponential decay regression curve is marked by a grey line in A—D (t_1/2_: (A) 3.7 days, (B) 5.3 days, (C) 2.5 days and (D) 3.5 days).

Tracer enrichments in the liver cytoskeletal (CYTO) and connective tissue (CT) proteins are presented as individual enrichments values with a curve indicating the mean enrichment in Figs 3 and 4, respectively. At all ages, an acute enrichment in both CYTO and CT tissue fractions is seen. Most of this enrichment disappears again within 30–40 days.

#### Muscle

**Fig 5:**
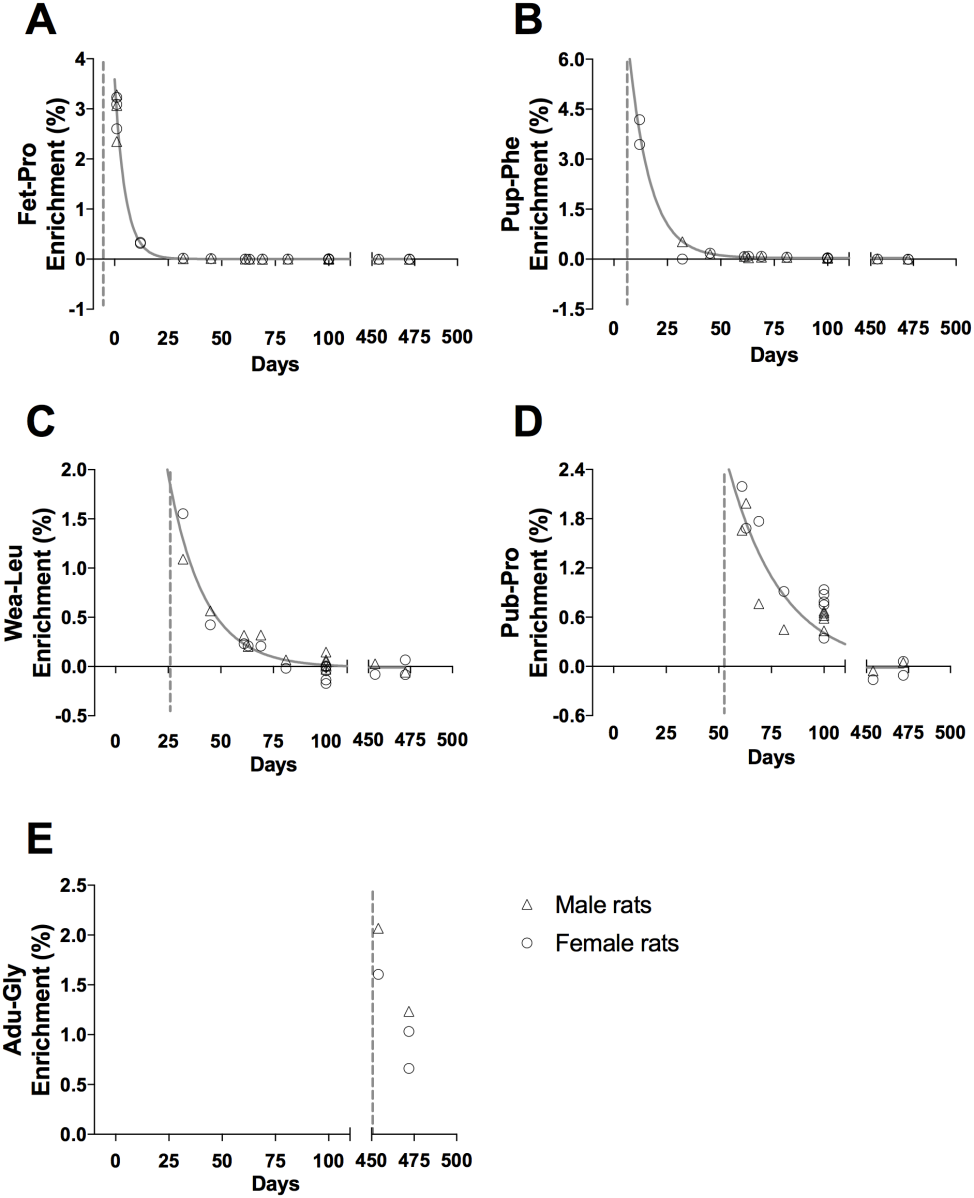
Tracer enrichment in muscle cytoskeletal/contractile proteins, CYTO. (A) Fet-Pro, (B) Pup-Phe, (C) Wea-Leu, (D) Pub-Pro, (E) Adu-Gly. Male (triangle) and female rat (circle) values are represented as individual enrichment values (%) in the muscle CYTO. The dashed vertical line indicates the start of the injection period of the tracer. An exponential decay regression curve is marked by a grey line in A—D (t_1/2_: (A) 3.5 days, (B) 6.9 days, (C) 11.8 days and (D) 17.9 days).

**Fig 6:**
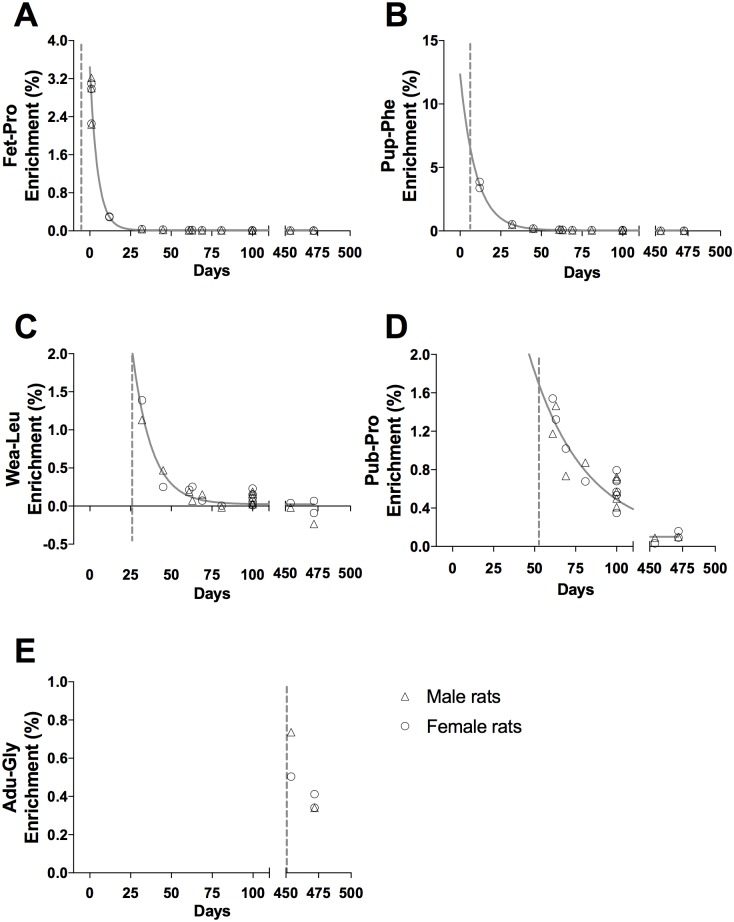
Tracer enrichment in muscle connective tissue proteins, CT. (A) Fet-Pro, (B) Pup-Phe, (C) Wea-Leu, (D) Pub-Pro, (E) Adu-Gly. Male (triangle) and female rat (circle) values are represented as individual enrichment values (%) in the muscle CT. The dashed vertical line indicates the start of the injection period of the tracer. An exponential decay regression curve is marked by a grey line in A-D (t_1/2_: (A) 3.3 days, (B) 6.8 days, (C) 8.3 days and (D) 23.3 days).

Muscle proteins behave similar to liver proteins. At all ages an acute enrichment in both CYTO (Fig 5) and CT (Fig 6) tissue fractions is seen and most disappears again within 30–50 days.

#### Eye lens

**Fig 7:**
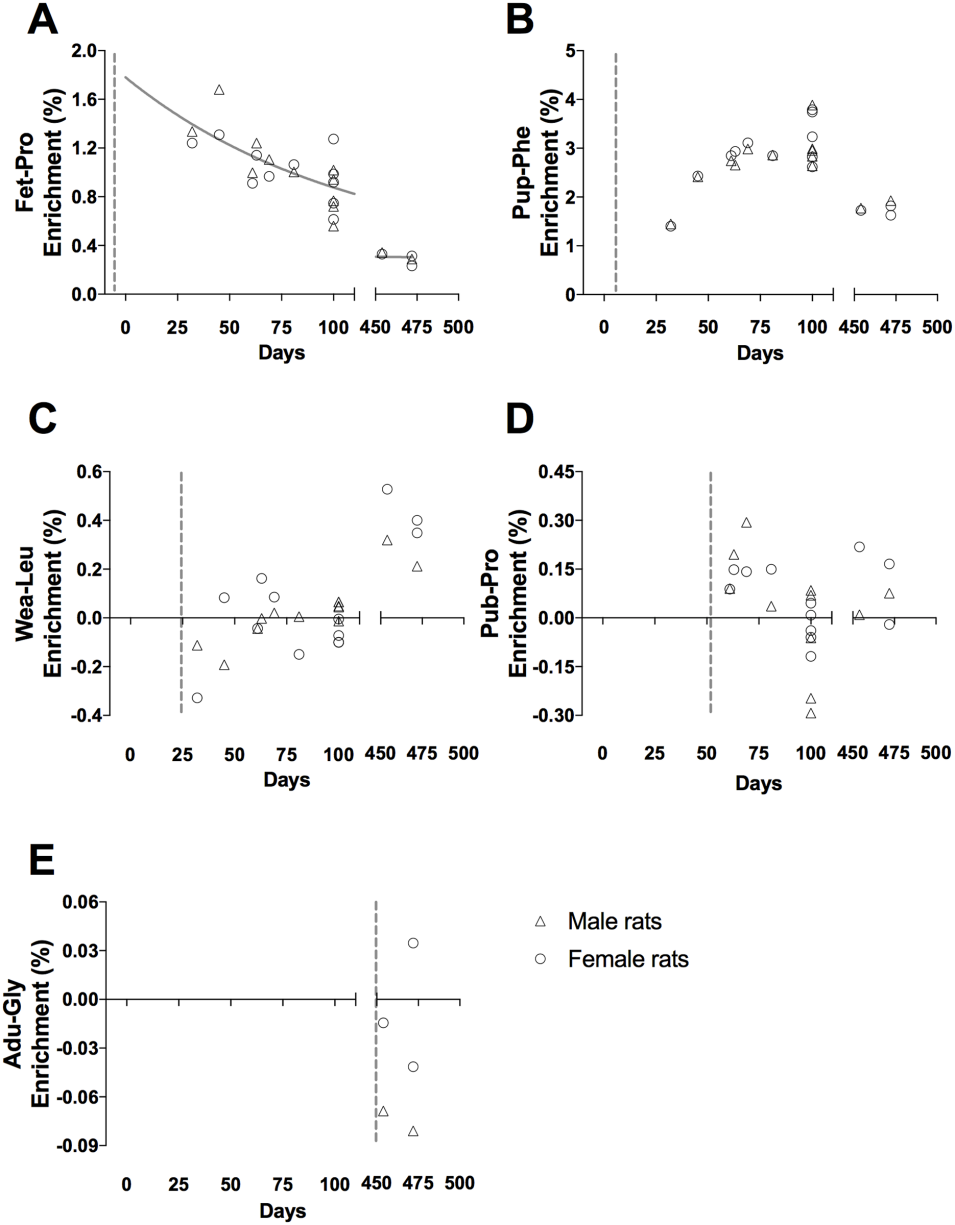
Tracer enrichment in eye lens connective tissue proteins, CT. (A) Fet-Pro, (B) Pup-Phe, (C) Wea-Leu, (D) Pub-Pro, (E) Adu-Gly. Male (triangle) and female rat (circle) values are represented as individual enrichment values in the eye lens CT. An outlier on 3.46% enrichment was removed from Wea-Leu (Fig. 7C, n = 9) at day 100. The dashed vertical line indicates the start of the injection period of the tracer. An exponential decay regression curve is marked by a grey line in A (t_1/2_: (A) 74.7 days).

A completely different picture is seen in the eye lens. The early Fet-Pro and Pup-Phe tracers in eye lens CT fraction are still present even at day 472 (Fig 7). In contrast, after day 54 the incorporation of tracers is very limited (Wea-Leu and Pub-Pro) or completely absent (Adu-Gly).

#### Patellar tendon

**Fig 8:**
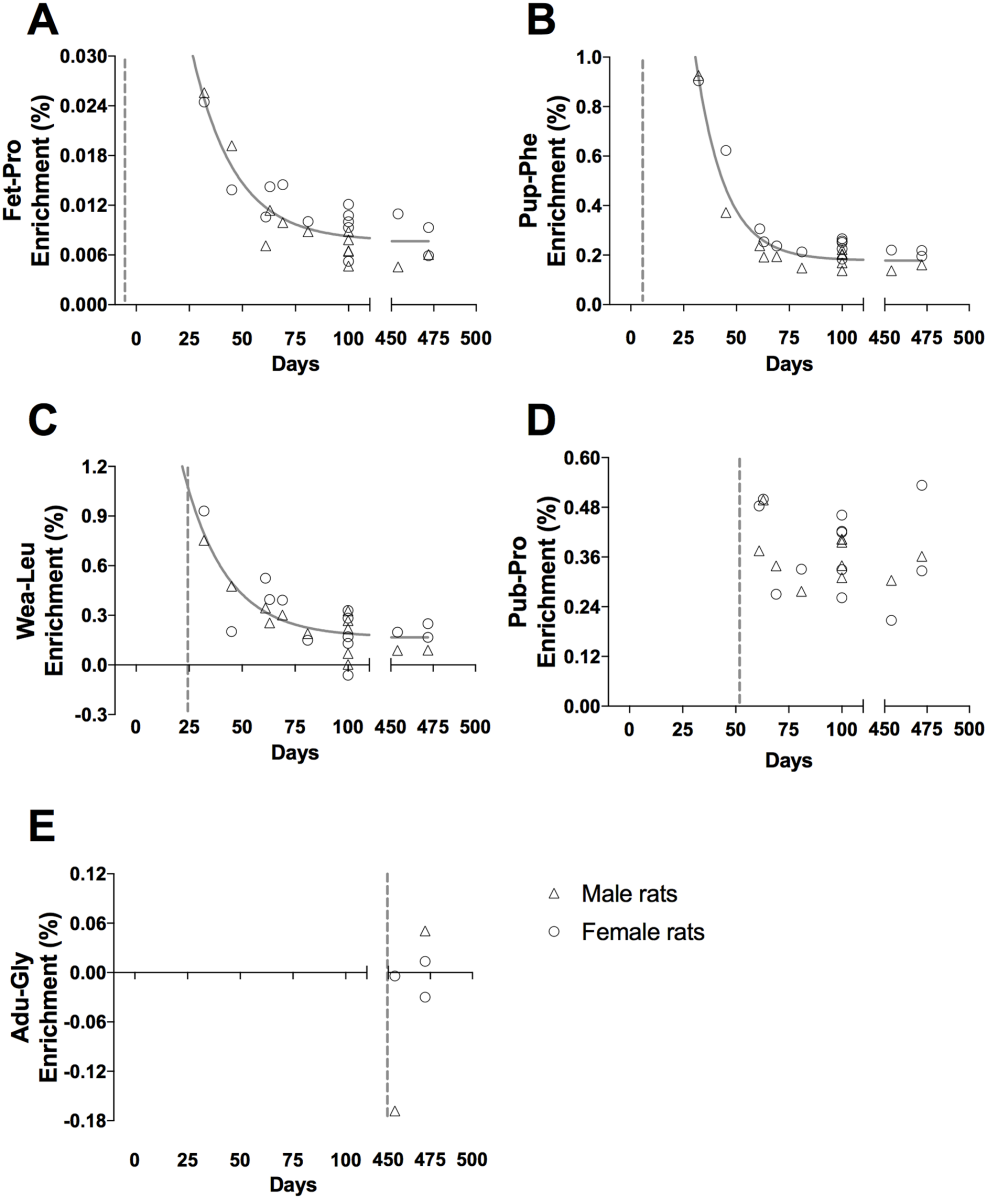
Tracer enrichment in patellar tendon connective tissue proteins, CT. (A) Fet-Pro, (B) Pup-Phe, (C) Wea-Leu, (D) Pub-Pro, (E) Adu-Gly. Male (triangle) and female rat (circle) and values are represented as individual enrichment values (%) in the patellar tendon CT. The dashed vertical line indicates the start of the injection period of the tracer. An exponential decay regression curve is marked by a grey line in A-C (t_1/2_: (A) 14.0 days, (B) 9.6 days, (C) 14.2 days).

The enrichment profile for patellar tendon CT proteins (Fig 8) appears to be a mix of the fast acute liver/muscle decrease in enrichment and the persistent enrichment seen in the eye lens, clearly indicating the existence of two pools. The Fet-Pro, Pup-Phe, Wea-Leu and Pub-Pro tracers enrichment drops fast within the first weeks, but then remains more or less constant and are still present at day 472. In the old rats, the incorporation of tracer is completely absent (Adu-Gly).

### Tracer enrichments at day 100

**Fig 9:**
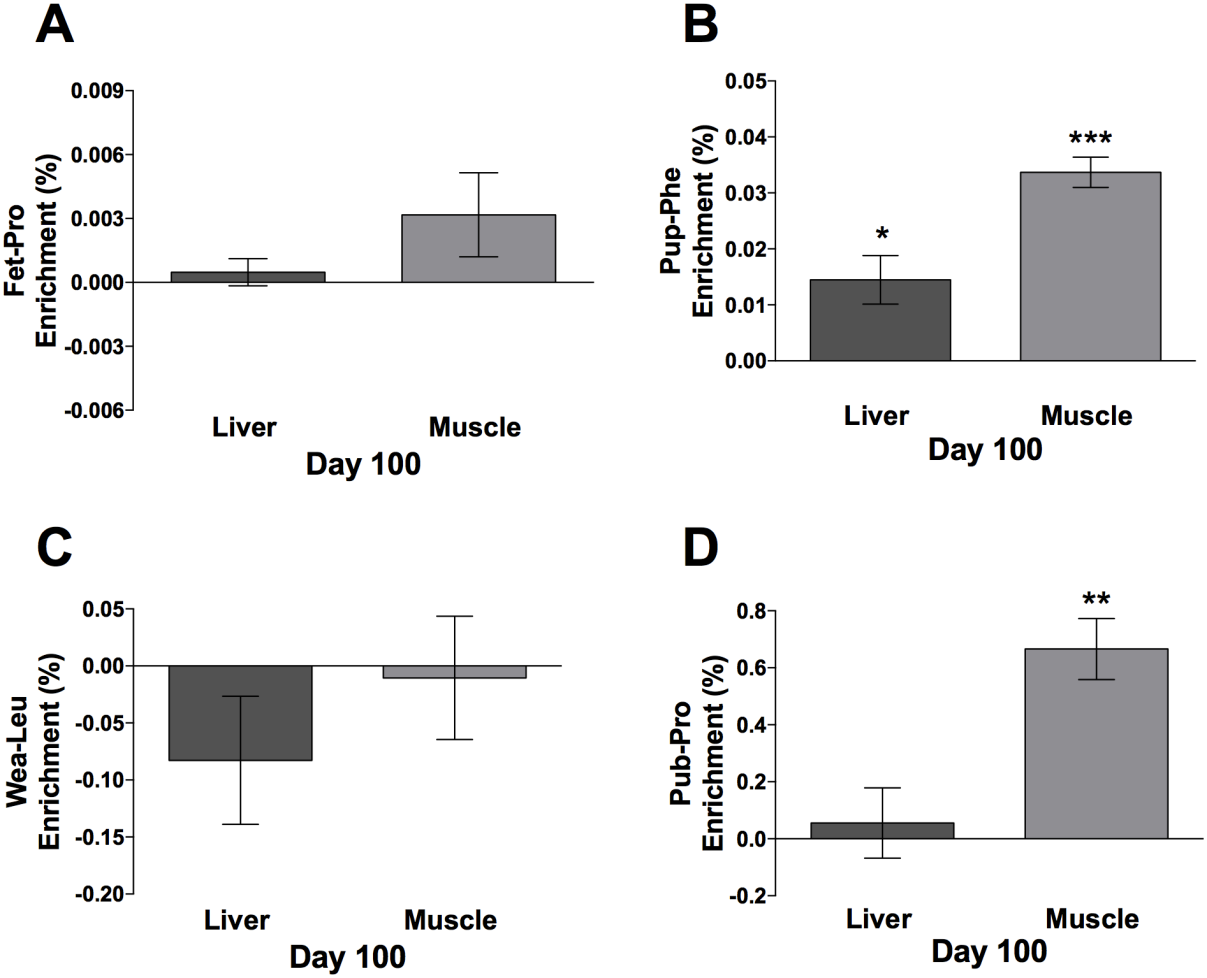
Day 100 tracer enrichment in cytoskeletal/contractile proteins, CYTO. (A) Fet-Pro, (B) Pup-Phe, (C) Wea-Leu, (D) Pub-Pro. Values are represented as enrichment (%), mean ± 95% CI analyzed in the liver and muscle CYTO, n = 10. *p < 0.05, **p < 0.01, ***p < 0.001.

At day 100, ten rats were euthanized to facilitate statistical test of retained enrichment. CYTO enrichment data from the liver and muscle are presented as mean ± CI (n = 10) in Fig 9. At the age of 100 days it was not possible to find the Fet-Pro (Day -10 to -2) tracer in muscle and liver. However, the Pup-Phe (Day 5–9) tracer was still present in both the liver (*p* < 0.05) and muscle (*p* < 0.001), with twice as much enrichment in muscle as in liver. The Leu-Wea (Day 25–29) tracer was not present in neither the liver nor the muscle. Pub-Pro (Day 54–58) tracer was only present in the muscle (*p* < 0.01).

**Fig 10:**
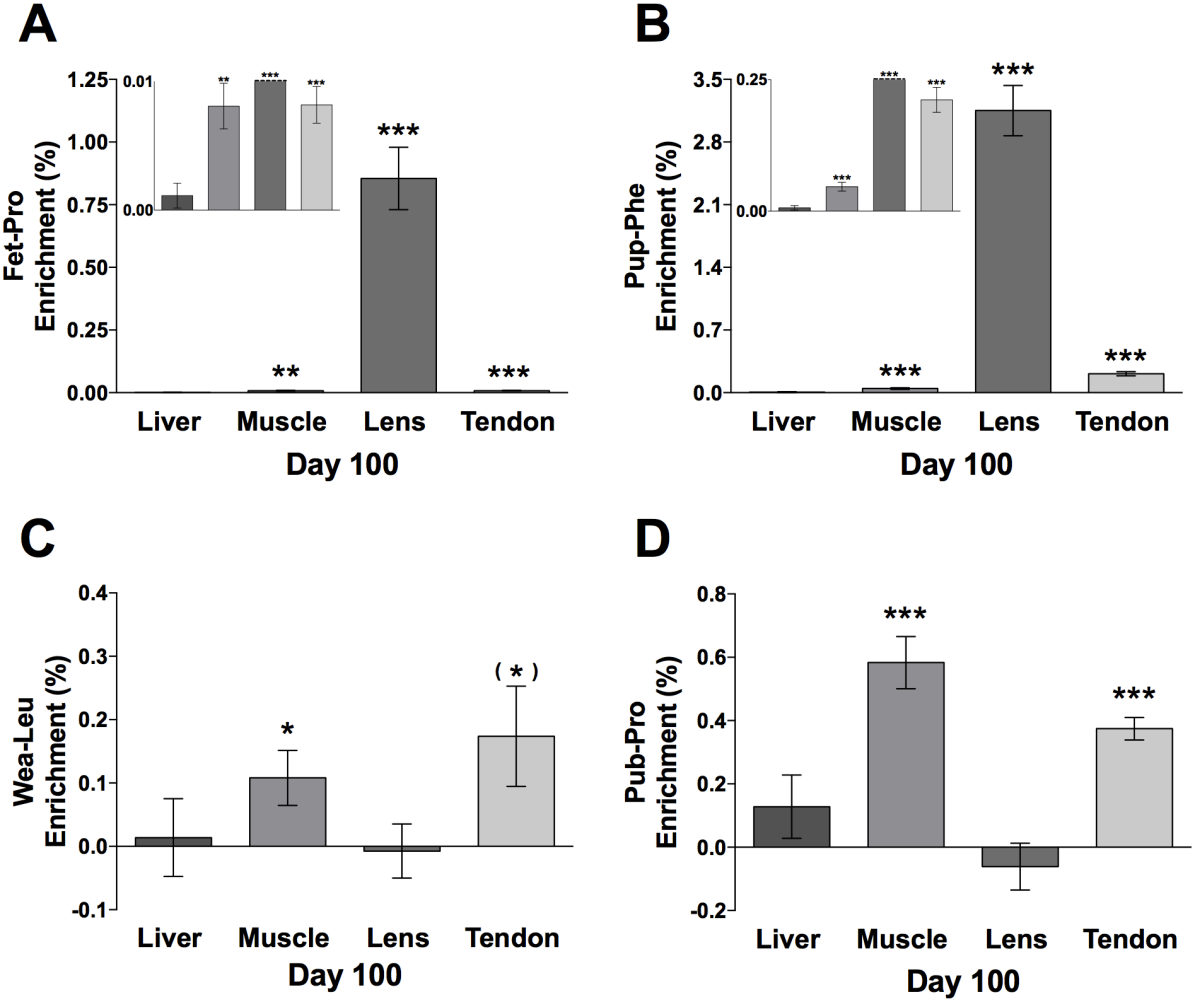
Day 100 tracer enrichment in connective tissue proteins, CT. (A) Fet-Pro, (B) Pup-Phe, (C) Wea-Leu, (D) Pub-Pro. Values are represented as enrichment (%), mean ± 95% CI analyzed in the CT in liver, muscle, eye lens (one outlier of 3.46% enrichment was removed, Fig. 10C) and patellar tendon, n = 10. *p < 0.05, **p < 0.01, ***p < 0.001. Fig. 10A and 10B includes inserts showing the distribution of the enrichment at baseline. The orders of the tissues are the same as the large panels.

CT enrichment data from the liver, muscle, eye lens and patellar tendon are presented as mean ± CI in Fig 10 (n = 10). All tissues except liver were still enriched for the Fet-Pro (Day -10 to -2); muscle (*p* < 0.01), eye lens (*p* < 0.001) and patellar tendon (*p* < 0.001), with eye lens far more enriched (0.9%) than the other tissues (0.001–0.008%). Similar data were obtained for the Pup-Phe (Day 5–9) tracer, which was also found in all tissues except liver; muscle (*p* < 0.001), eye lens (*p* < 0.001) and patellar tendon (*p* < 0.001). Still eye lens had much higher enrichment (3.2%) than the other tissues. However, the range between the other tissues was larger; liver 0.006% (not significant), muscle 0.05% and patellar tendon 0.19%. In contrast, the Leu-Wea (Day 25–29) tracer was not retained in the eye lens (or liver) but was present in the muscle (*p* < 0.05) and patellar tendon (tendency, *p* = 0.057) at comparable levels. Similarly, the Pub-Pro (Day 54–58) was found in both muscle (*p* < 0.001) and patellar tendon (*p* < 0.001), but not in the eye lens and liver.

### Tracer enrichments at day 472

**Fig 11:**
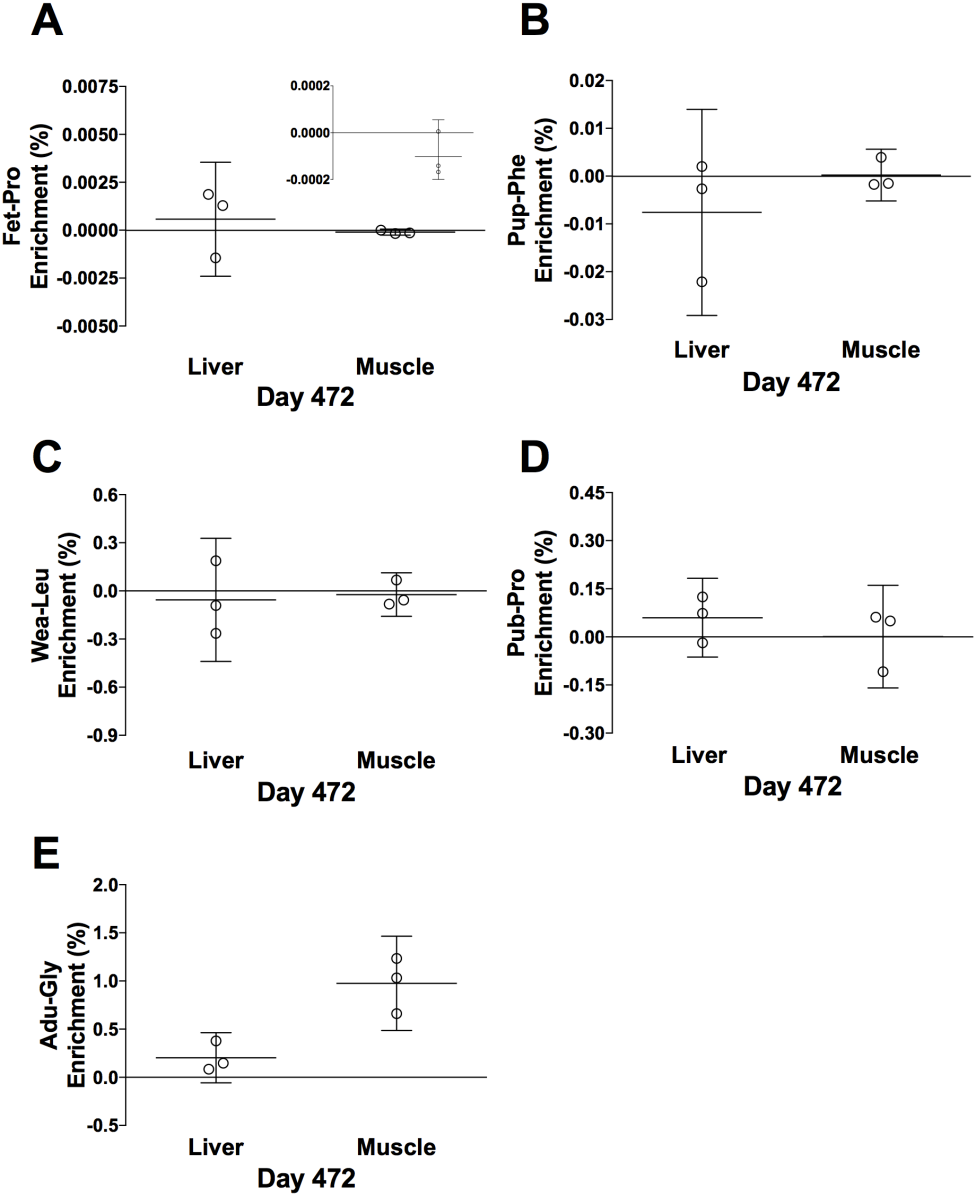
Day 472 tracer enrichment in cytoskeletal/contractile proteins, CYTO. (A) Fet-Pro, (B) Pup-Phe, (C) Wea-Leu, (D) Pub-Pro, (E) Adu-Gly. Values are represented as individual enrichments (%), mean ± 95% CI analyzed in the liver and muscle CYTO, n = 3. Fig. 11A includes an insert showing the distribution of the enrichment at baseline. The orders of the tissues are the same as the large panels.

**Fig 12:**
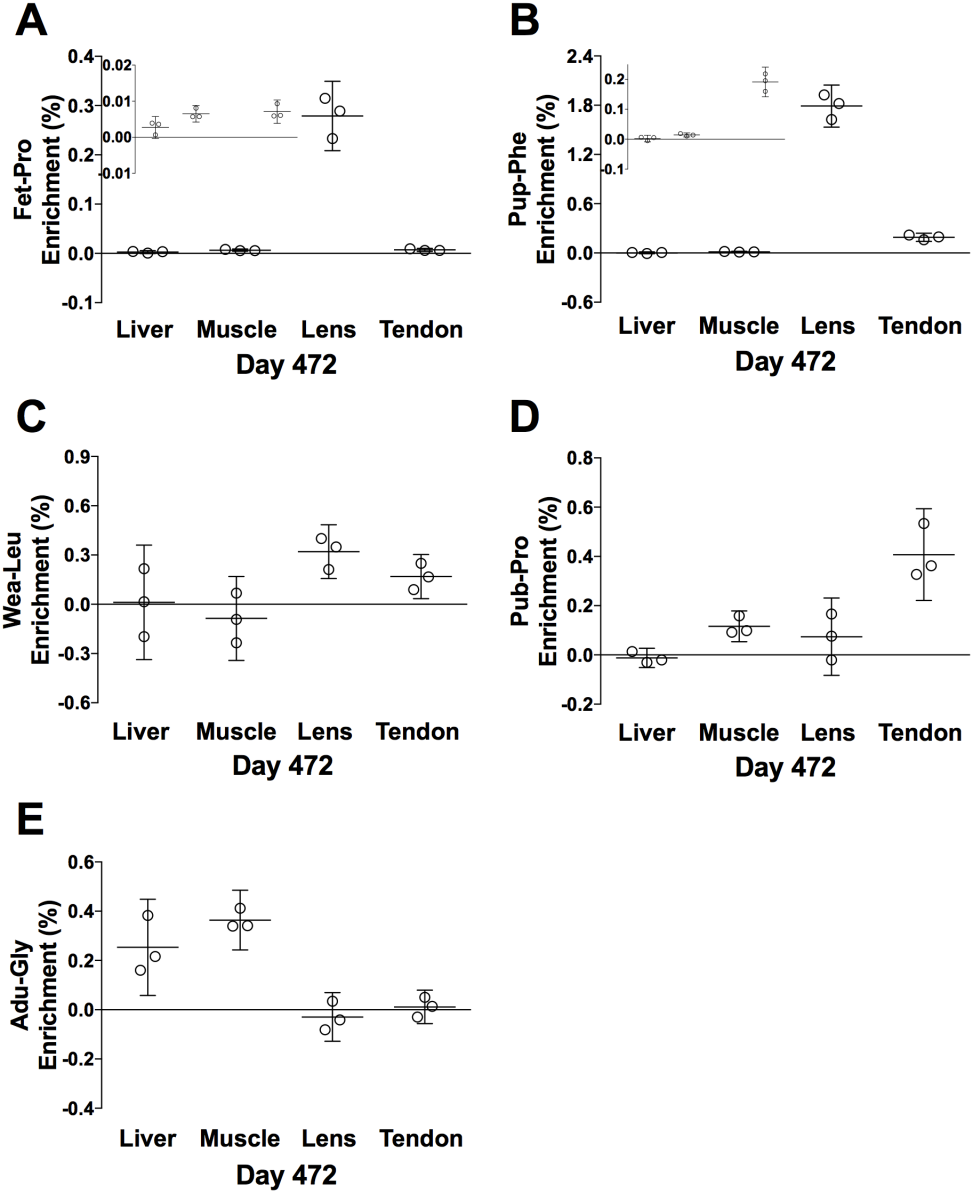
Day 472 tracer enrichment in connective tissue proteins, CT. (A) Fet-Pro, (B) Pup-Phe, (C) Wea-Leu, (D) Pub-Pro, (E) Adu-Gly. Values are represented as individual enrichments (%), mean ± 95% CI analyzed in the liver, muscle, eye lens and patellar tendon CT, n = 3. Fig. 12A and 12B includes inserts showing the distribution of the enrichment at baseline. The orders of the tissues are the same as the large panels.

Three rats were kept alive for ~ 16 months to let them grow old. Before euthanization at day 472, they were injected with a fifth tracer (Adu-Gly). CYTO enrichment data from the liver and muscle are presented in Fig 11. Compared to day 100 (Fig 9), neither of the earlier tracers seems to be retained at day 472. Only the last tracer (Adu-Gly, Day 447–451) appeared to be present in the muscle and perhaps liver (compare Figs 11E and 12E). CT enrichment data from all tissues are presented in Fig 12. Even at the age of 472 days, the Fet-Pro (Day -10 to -2) tracer was still clearly measurable in the eye lens and perhaps also at low level in other tissues. The Pup-Phe (Day 5–9) tracer was also retained in the eye lens and perhaps at low level in the patellar tendon and muscle, but not in liver. The Wea-Leu (Day 25–39) tracer seems only to have been retained in eye lens and patellar tendon. However, the Pub-Pro (Day 54–58) tracer seems also present in the muscle besides patellar tendon and perhaps eye lens. In contrast, at the old age the Adu-Gly (Day 447–451) tracer was only captured in the liver and muscle but not in the eye lens and the patellar tendon.

## Discussion

The primary aim of this study was to investigate whether we could demonstrate the existence of protein pools in different tissues and within the same tissue with very distinct turnover rates in an *in vivo* rodent model. Of most interest, we first demonstrate that amino acids synthesized into proteins during fetal development and first weeks of life are present in the eye lens and patellar tendon tissue at adult age. Secondly, we demonstrate in the eye lens that there is no detectable transfer of newly synthetized proteins into mature structural connective tissue beyond the age of 25 days despite an acute incorporation of tracers into other tissues’ proteins. This age-dependent growth of eye lens tissue is in contrast to proteins located in the muscle and liver, which showed a fast turnover independent of age.

### Turnover of collagenous proteins from various tissues

The primary aim was to investigate the turnover of collagenous tissue proteins throughout life and therefore, we collected eye lens, patellar tendon, skeletal muscle and liver tissues from rats. The latter tissue also contained protein pools expected to contain fast turning over proteins that could be used to verify our approach as being adequate to elucidate the aim. The eye lens and tendon showed a marked detainment of amino acid tracer abundance throughout age compared to the structural proteins from liver and muscle. In the eye lens, tracers injected early in life, both during the fetus development and the first post-natal period (day 5–9) were still present at day 472. In addition, we did not detect any immediate incorporation of tracers beyond the age of 25 days. Also for the Wea-Leu and Pub-Pro tracers we could not detect enrichments at day 100. However, in the adult rats at day 450 and 472 we found enrichments in all (Wea-Leu) or in some (Pub-Pro) samples, which though indicate that there may be parts of the eye lens that undergo remodeling later in life. Another indication of an ongoing remodeling process of the eye lens is the drop in enrichment of Fet-Pro and Pup-Phe tracers from day 100 to day 450/472. Hence, based on these results it may be suggested that the remodeling process of the matrix in the eye lens takes place involving different steps than the classic immature/soluble-to-mature/insoluble process [21, 22]. However, we conclude that more research must be conducted to verify the observations and reveal the complexity of the collagen synthesis and remodeling in the eye lens during adult life in rats, since the data on day 100 are conclusive and the sample size at adult age is less and data more inconsistent. Hence, in accordance with results from human eye lens, where Lynnerup et al. demonstrated that no further proteins were synthesized and incorporated into the lens matrix after the age of 1 year [1] our data (to some extend) support this finding in rats.

With our repeated injections of different tracers early in life, we could further demonstrate that even proteins synthesized during fetal development and 5–9 days of age are present in the eye lens at the age of day 100 and 472 (Figs 10A, 10B, 12A and 12B). Though, a decline in the enrichment of all tracers (given at day -10 to -2, day 5–9 and day 25–39, Figs 7A, 7B, 8A and 8C) compared to the enrichment detected at day 61–100 and day 454–472 (Figs 7D, 7E, 8D and 8E) was found. While this decline can be a result of partly loss of protein-bound labeled amino acids due to degradation of labeled proteins, and partly as a consequence of growth, i.e. a ‘growth dilution’ [23]. That is, that the tracer is not lost due to breakdown of proteins, but the tracer enrichment in the tissue proteins is diluted due to the incorporation of unlabeled ‘tracee’-amino acids into proteins synthesized later in life. This phenomenon is supported by the fact that at the subsequent ages (fetal development and pup age) the tracers were actually incorporated into the proteins revealing a growth at these subsequent ages. However, beyond the age of 25 days we could not detect any synthesis of proteins in the eye lenses and hence, no further growth dilution can be expected beyond this age.

The patellar tendon showed incorporation of tracers at the stages; fetus, pup, weaning and puberty (i.e. the first four tracers (Fig 8A–8D)) meaning growth up till the age of 60 days. At day 472 it was not possible to detect the presence of the last injected tracer (injected day 447–451) in the tendon proteins. Therefore, it can be concluded that the rat tendon turns inert and refrain from exchanging amino acids somewhere between day 60 and 472. However, the tracer incorporated at day 55–59 was present in the same ratio at day 100 and 472. Therefore, it is unlikely that significant amounts of proteins are lost and added to the tissue beyond the age of 60 days. Hence, the growth and turnover of tendon proteins are presumably to have ceased shortly after the age of 60 days. As a further emphasis on this growth cease around the age of 60 days is that none of the first three tracers’ abundance are diluted in the tendons proteins beyond that age, suggesting no further addition of unlabeled amino acids, i.e. growth, after that age. In contrast, all the tracers given during fetal development and day 5–9 and 20–25 are diluted markedly from day 25 to 50. The initial decline could be due to the ‘growth dilution’. However, it could also be an example of the complexity of the growth of the tendon structure. Different studies have shown very contrasting turnover rates; ranging from hours [9] to years [2] and intermediate rates [24] in human tendon. Similar results on collagen turnover in rats using the same techniques, have, to our knowledge, not been published. But an old radioactive tracer-labeling experiment showed slow turnover of collagen proteins in rat tendon with age [23]. Furthermore, a study measuring the breakdown of collagenous proteins in rats has shown the same slow turnover [25]. In humans, it has been shown that the core of a human patellar tendon is developed during the first 17 years of age, and that the core tendon proteins hereafter are maintained during adulthood [2]. As other studies have been able to detect incorporation of amino acid tracers into collagenous patellar tendon tissue within few hours [9], these findings taken together suggest that tendon tissue contains distinct pools of proteins; one that continuously synthesizes proteins and is sensitive to strain (thigh muscle contractions [24] and even nutrition [26] (maybe located as a superficial layer)) and an inert pool of proteins (maybe making up the majority of the tendon core). Thus, the initial decline in enrichment, or rather the very first spike in enrichment, may be a consequence of a protein pool that inhere a fast turnover where proteins are synthesized de novo but only partly (or not at all) matured and used for structural growth and thus is mainly degraded again, leaving the later enrichment far lower than the initial spike. This result is also seen in another study, where the turnover of soluble (fast) and insoluble (slow) collagen proteins from skin on rats where examined [27]. In that study 25 days old rats were exposed to atmospheric ^18^O_2_ for 36 hours, and skin samples were collected from several time points until 392 days after exposure. The initial peak of the soluble (fast) collagen pool faded out after 50 days, followed by an almost constant enrichment until the end at day 392. Also a study done on mice, which was used to examine lung fibrosis, showed a faster synthesis rate of soluble extra cellular matrix proteins compared to insoluble extra cellular matrix proteins, which reflected more mature matrix components seen in the insoluble pool [28]. This corresponds well with our findings with a fast declining peak followed by a long stable period of enrichment in the patellar tendon, where newly synthesized collagenous proteins are built into mature highly cross-linked structures forming the in-soluble functional matrix characterizing healthy tendons [21, 29].

In both liver and skeletal muscle the connective tissue collagenous proteins showed a markedly larger incorporation of tracer immediately after injections than the eye lens and tendon (peak enrichments in Figs 4 and 6 compared to Figs 7 and 8). This high immediate enrichment, which is comparable to that in the cytoskeletal protein fraction from the same tissues, reflects that liver and muscle collagenous protein has (at least a pool) of proteins with high synthesis rate. This corresponds to the synthesis rate of human muscle collagenous connective tissue that seems as high as myofibrillar proteins [13, 30]. The rapid decrease of the abundance of the tracer (Figs 4 and 6) reveals that the connective tissue matrix consists of collagenous proteins that continuously undergo turnover and that no proteins remain in these tissues for more than 50 days. Although very similar to liver early in life, the muscle connective tissue (similar to the cytoskeletal proteins) may transfer part of the newly synthesized proteins into a more lasting pool of proteins as the enrichments remain longer in skeletal muscle compared to similar protein fractions in the liver (Fig 6C and 6D). In humans with fibrotic liver disease, liver collagenous proteins were found to turnover with a rate of two months to one year and to have a pool of newly formed collagen that got larger the more severe the disease condition was [22]. All in all, these data support the notion that in tissues with collagenous protein as a supportive structure (e.g. liver and muscle) the collagen pool is made up of a pool of immature and fast turning over collagen proteins that matures into larger and more insoluble and cross-linked matrix proteins with a slower turnover.

### Verification of tracer-labeling approach

We collected soleus muscle and liver tissues to obtain the myofibrillar and cytoskeletal protein fractions, respectively, that we used as control to verify our tracer labeling approach. From previous data these two protein fractions have a high synthesis rate throughout life even though they decrease with age [31, 32]. Determining the tracer abundances in these proteins shortly after tracer injections would therefore allow us to demonstrate that the repeated injections of different stable isotopically labeled amino acids at various ages was a feasible method to label tissue and structural proteins throughout age. In both muscle and liver cytoskeleton proteins, we detected marked incorporation of all used tracers two days after the last of five repeated injections. On the subsequent time points for tissue collection a significant loss of tracer was seen and 30–50 days after being injected all tracers had disappeared. These findings reveal a high turnover of these tissue-proteins-fractions and this finding is in agreement with other studies using isotope labeling of similar types of proteins within a short period of time relative to the animal’s lifespan [23, 33]. Therefore, we are confident that the labeling approach applied in this study was suitable for investigating temporal dynamics of the turnover of different proteins with special emphasis on collagenous connective tissues.

Using five different isotopically labeled amino acids as tracers gave us the opportunity to investigate whether protein fractions within the same tissue and from different tissues exhibit synthesis of proteins during the different time points of the rats’ life. This approach seemed to work in the present experiment, as all the tracers were detectable in some proteins after the injection periods. Our setting and the applied analyses were of cause limited by analytical sensitivity, which varied dependent on the number of labeled atoms on the chosen amino acid tracers. Hence, a theoretical limitation of the study is that it may be likely that the exposure to one or more tracers during the injection period may have been insufficient to allow detectable tracer to be incorporated into very slow turning over proteins. This we believe is though theoretical as the tracer exposure periods were temporally extensive in the perspective of the rats’ life and we applied rather large tracer amounts. Further, we selected several tracers with multiple labeled atoms, which improved the analytical sensitivity making us able to determine lower enrichments of these tracers. Therefore, we believe that we would have detected incorporation of most exposed tracers in any protein pool with a physiologically relevant turnover rate and likewise preservation of the tracers in very slow turning over or inert proteins.

The observed variation on the enrichments of the tissues/proteins at individual time points is caused by several factors and can best be evaluated at day 100 due to the number of measured animals at this time point. The variation in tracer abundances is made up by differences in the precursor tracer enrichment, which are decisive for the protein-bound tracer abundance. Although, we injected tracers per kg body weight, some inter-animal differences must be acknowledged, hence, resulting in some variation between animals at the subsequent time points. Further, also biological variations between and within the animals consisting of i) inter-animal differences in turnover rates and ii) tissue specimen site differences, is presumably also responsible for the observed variation. These biological variations are relative, range of coefficient of variation [15–30%], and are therefore grossly unaffected by the level of enrichment. Finally, also analytical reproducibility is present, however, it is estimated to contribute less to the reported gross variation.

Finally, a potential problem of using different amino acids as tracers is that they in theory could act differently during protein synthesis at the different time points. However, since the aim of the experiment was incorporation of the tracer during growth, and not a quantification of the protein synthesis or degradation rates, the experimental setup seemed to be useful for our main purpose.

## Conclusion

We here demonstrate that it is possible to label proteins early in life through frequent tracer injections using stable isotopically labeled amino acids, and find them in the eye lens and patellar tendon of the mature rodent. The results from the eye lens and to some extent the patellar tendon demonstrate possible existence of inert proteins, in contrast to proteins located in the muscle and liver, which showed a fast turnover, independent of age. Secondly, we demonstrate no detectable transfer of newly synthetized proteins into mature structural connective tissue beyond the age of 25 days despite an acute incorporation of tracers into tissue proteins.

The data support the hypothesis that some proteins synthesized during the early development and growth are still present later in life in animals and that divergent results on the synthesis rates of collagenous proteins are evenly right, though just reflect different protein pools.
